# Morphology-Controlled Synthesis of Polyphosphazene-Based Micro- and Nano-Materials and Their Application as Flame Retardants

**DOI:** 10.3390/polym14102072

**Published:** 2022-05-19

**Authors:** Yuanzhao Zhu, Wei Wu, Tong Xu, Hong Xu, Yi Zhong, Linping Zhang, Yimeng Ma, Xiaofeng Sui, Bijia Wang, Xueling Feng, Zhiping Mao

**Affiliations:** 1 Key Laboratory of Science and Technology of Eco-Textile, Ministry of Education, College of Chemistry, Chemical Engineering and Biotechnology, Donghua University, Shanghai 201620, China; 1189171@mail.dhu.edu.cn (Y.Z.); wuwei@dhu.edu.cn (W.W.); xutong.868@163.com (T.X.); hxu@dhu.edu.cn (H.X.); zhongyi@dhu.edu.cn (Y.Z.); zhang_lp@dhu.edu.cn (L.Z.); yimeng.ma@dhu.edu.cn (Y.M.); suixf@dhu.edu.cn (X.S.); bwang@dhu.edu.cn (B.W.); xlfeng@dhu.edu.cn (X.F.); 2 Innovation Center for Textile Science and Technology, Donghua University, Shanghai 201620, China; 3 National Engineering Research Center for Dyeing and Finishing of Textiles, Donghua University, Shanghai 201620, China; 4 National Manufacturing Innovation Center of Advanced Dyeing and Finishing Technology, Donghua University, Shanghai 201620, China

**Keywords:** morphology-controlled, polyphosphazene, nano-materials, flame retardant, PET

## Abstract

Common flame retardants, such as halogen-based materials, are being phased-out owing to their harmful environmental and health effects. We prepared poly-(cyclotriphosphazene-co-4,4′-sulfonyldiphenol) (PZS) microspheres, nanotubes, capsicum-like nanotubes, and branched nanotubes as flame retardants. An increase in reaction temperature changed the morphology from nanotubes to microspheres. A PZS shape had a positive effect on the flame retardancy of polyethylene terephthalate (PET). The PZS with a capsicum-like nanotube morphology had the best flame retardancy, and the PET limiting oxygen index increased from 25.2% to 34.4%. The flame retardancy capability was followed by PZS microspheres (33.1%), branched nanotubes (32.8%), and nanotubes (32.5%). The capsicum-like nanotubes promote the formation of highly dense and continuous carbon layers, and they release a non-combustible gas (CO_2_). This study confirms polyphosphazene-based flame retardants as viable and environmentally-friendly alternatives to common flame retardants. It also presents a novel and facile design and synthesis of morphology-controlled nanomaterials with enhanced flame retardant properties.

## 1. Introduction

Hexachlorocyclotriphosphazene (HCCP) is a stable six-membered ring structure comprising alternating backbones of phosphorus and nitrogen atoms with sufficient thermal stability. Polyphosphazenes have a flexible molecular design and are excellent materials used in many fields, including flame retardance [1,2], drug delivery [3], optoelectrical materials [4,5], and catalysis [6]. Although halogen-based flame retardants are widely used, burning them releases a lot of harmful substances that may be carcinogenic and are hazardous to the environment; thus, they are being gradually restricted [7,8]. Polyphosphazene flame retardants are emerging as promising alternatives owing to their environmentally-friendly nature, which has stimulated considerable research interest.

In recent decades, research on controlling the morphology and size [9,10] of micro- and nano-materials has received significant attention. Many researchers [11,12,13] have selected HCCP as a monomer to react in tetrahydrofuran (THF), acetonitrile, N, N-dimethylformamide (DMF), and pyridine, using morphology-controlled methods for the synthesis of different micro- and nano-materials, such as microspheres, nanotubes, and nanofibers. Zhu et al. [13] reported a one-pot fabrication of poly-(cyclotriphosphazene-co-4,4′-sulfonyldiphenol) (PZS) nanotubes from the reaction of HCCP and 4,4′-sulfonyldiphenol (BPS) in THF at room temperature. Triethylamine (TEA) was used as an acid acceptor to absorb the hydrochloric acid (HCl) generated during the reaction. Zhu et al. [14] further reported a one-pot fabrication of monodispersed PZS microspheres in a room temperature reaction of HCCP and BPS in the presence of TEA in acetone. Fu et al. [15] successfully synthesized capsicum-like PZS nanotubes by slowly adding a toluene solution containing HCCP to an acetone solution containing BPS and TEA at room temperature. Fu et al. [16] successfully prepared branched PZS nanotubes using a two-step method, wherein small amounts of TEA were added to the HCCP and BPS reaction solution. The remaining TEA was added after 2 h, and the reaction was then allowed to continue for 10 h. Although many researchers have prepared polyphosphazene compounds with different morphologies using HCCP and BPS as reactants in different solutions, few have investigated how the polyphosphazene morphology affects flame retardancy.

In this study, four different morphologies of PZS polymers were prepared by adding reactants dropwise in THF and varying the reaction temperature. This study also aimed to elucidate the formation mechanism of PZS with different morphologies. To explore the effect of the PZS morphology on flame retardancy, the four different flame retardants were melt-blended with polyethylene terephthalate (PET). Lastly, the thermal stability, flame retardancy and combustion behavior, pyrolysis products, gas, and condensed phase flame retardant mechanism of the synthesized flame retardant–PET composites were analyzed.

## 2. Experimental

### 2.1. Materials

In this study, hexachlorocyclotriphosphazene (HCCP, 99%) and 4,4′-sulfonyldiphenol (BPS, 99.8%) were procured from Adamas Reagent Co., Ltd. (Shanghai, China). Ethanol (95%), THF (99.5%), and TEA (99.0%) were procured from General-Reagent. PET chips were procured from Sinopec Yizheng Chemical Fiber Co., Ltd. (Yizheng, China). All reagents were used as received.

### 2.2. Synthesis of Micro/Nanoscale PZSs

**Figure 1:**
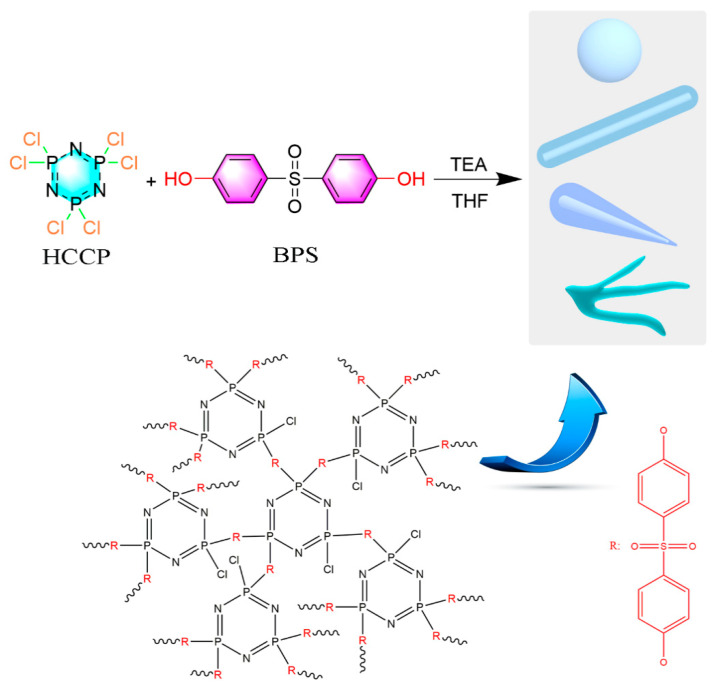
Scheme of the synthesis route of PZS with different morphologies and the proposed chemical structure.

The facile synthesis of PZS polyphosphazene flame retardants with different morphologies was conducted as set out below (Figure 1).

#### 2.2.1. Preparation of PZS Nanotubes (PZS_NT)

First, 0.2 g HCCP and 0.432 g BPS were dissolved in 200 mL THF in a three-necked flask; thereafter, 3 mL TEA was added to the above solution and the resulting reaction solution was mechanically stirred at room temperature for 3 h. The solution was then centrifuged, and the resulting precipitate was washed three times with ethanol and deionized water. The precipitate was freeze-dried at −50 °C for 24 h, followed by drying at 60 °C for 12 h to obtain a final powdered product.

#### 2.2.2. Preparation of PZS Microspheres (PZS_SP)

The preparation of PZS_SP followed the same procedure as that of PZS_NT presented in Section 2.2.1 above, except that the reaction was performed at 120 °C.

#### 2.2.3. Preparation of PZS Capsicum-like Nanotubes (PZS_CLNT)

First, 0.432 g BPS and 3 mL TEA were added to 150 mL THF in a three-necked flask (solution A). Thereafter, 0.2 g HCCP was dissolved in 50 mL THF (solution B). Solution B was slowly added dropwise to solution A under mechanical stirring for 2 h. The reaction was conducted at room temperature for 3 h. The post-synthesis procedures of centrifugation and drying were performed according to the procedure for PZS_NT presented in Section 2.2.1 above.

#### 2.2.4. Preparation of PZS Branched Nanotubes (PZS_BNT)

First, 0.432 g BPS and 3 mL TEA were added to 150 mL THF in a three-necked flask (solution A). Thereafter, 0.2 g HCCP was dissolved in 50 mL THF (solution B). Half of solution B was slowly added dropwise into solution A over a period of 1 h under mechanical stirring at room temperature. After 1 h, the other half of solution B was also slowly added dropwise over a period of 1 h and the reaction was allowed to continue for 2 h. The post-synthesis procedures of centrifugation and drying were performed according to the procedure for PZS_NT presented in Section 2.2.1 above.

### 2.3. Preparation of PET/PZS Composites

PET chips were crushed and mixed with each of the different morphologies of PZS flame retardants (5 wt%). To remove moisture, the mixed samples were placed in an oven at 140 °C for 12 h. The samples were then processed using a two-screw extruder (WLG10G, Xinshuo Precision Machinery Co., Ltd., Suzhou, China) at 260 °C, at a screw speed of 40 rpm. Finally, the PET/PZS flame retardant composites, with varying sizes, were subjected to injection molding according to testing standards using a precision micro-injection molding machine (Suzhou Yangyi Vouch Testing Technology Co., Ltd., Suzhou, China) at 245 °C in segment 1, 245 °C in segment 2, and 230 °C in segment 3, at a screw speed of 40 rpm.

### 2.4. Characterization Techniques

Fourier transform infrared (FTIR) spectra were measured on a PerkinElmer Spectrum Two spectrometer (Germany) over the wavenumber range of 400–4000 cm^−1^. X-ray diffraction (XRD) was conducted using an X-ray diffractometer (D/max-2550 PC, Tokyo, Japan) with Cu Kα radiation (λ = 0.15405 nm). Sample morphologies were obtained using scanning electron microscopy (SEM, Hitachi TM-3030, Japan) and transmission electron microscopy (TEM, JEM-2100, Tokyo, Japan). Thermogravimetric analysis (TGA, Netzsch TG209 F1 Libra, Netzsch, Germany) was recorded under an N_2_ atmosphere in the temperature range of 50–800 °C at a heating rate of 10 °C/min.

The limiting oxygen index (LOI) values were measured using a 5801A digital oxygen index analyzer (Suzhou Yangyi Vouch Testing Technology Co., Ltd., Suzhou, China) in accordance with the ISO 4589-2 testing standard. The dimension of each sample for the LOI test was 130 mm × 10 mm × 3.5 mm. Underwriter Laboratory 94 vertical burning tests (UL-94 V) were conducted using a vertical burning instrument (5402A, Suzhou Yangyi Vouch Testing Technology Co., Ltd., Suzhou, China) according to the ASTM D3801 testing standard, with spline dimensions of 130 mm × 13 mm × 3.2 mm. The combustion behavior of PET and PET/PZS composites was measured using a 6810-cone calorimeter (Suzhou Yangyi Vouch Testing Technology Co., Ltd., Suzhou, China) with sample sizes of 100 mm × 100 mm × 3 mm under a heat flux of 50 kW/m^2^ based on the ISO 5660-1 testing standard. Thermogravimetric analysis–Fourier transform infrared (TG-IR) spectra of the PET and PET/PZS composites were performed using a TA Q50 thermal gravimetric analyzer coupled with a Nicolet IS50 spectrometer (Madison, WI, USA) in the temperature range of 50–800 °C under an N_2_ atmosphere. Pyrolysis-gas chromatography-mass spectrometry (Py-GC/MS) was employed to analyze the pyrolysis products of PET/PZS flame retardant composites using a FRONTIER PY-2020iD Pyrolyser and a SHIMADZU GCMS QP2010 (Japan). Laser Raman spectroscopy of the char residue was conducted using a DXR2Xi laser Raman spectrometer (Thermo Fisher Scientific Co., Madison, WI, USA) equipped with an excitation wavelength of 532 nm, with measurements recorded in the range of 500–4000 cm^−1^.

## 3. Results and Discussion

### 3.1. Characterization of PZSs

**Figure 2:**
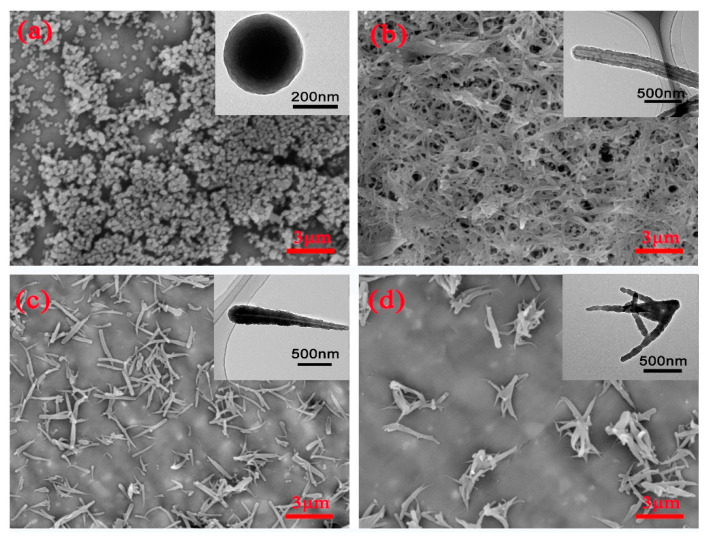
SEM and TEM images of PZS with different morphologies: (**a**) PZS microspheres, (**b**) PZS nanotubes, (**c**) PZS capsicum-like nanotubes, and (**d**) PZS branched nanotubes.

The microstructure of the fabricated PZS samples was characterized using SEM and TEM, and the results are shown in Figure 2 and Figure S1. Figure S1 reveals that PZS nanotubes were more easily formed at room temperature, whereas both PZS irregular nanotubes and microspheres were obtained at 80 °C. At 120 °C, the morphology changed to PZS microspheres. By adding the reactants dropwise, capsicum-like and branched PZS nanotubes were obtained. Lastly, PZS microspheres, nanotubes, capsicum-like nanotubes, and branched nanotubes were fabricated in THF (Figure 2). Notably, the diameter of the PZS microspheres was approximately 300 nm. PZS nanotubes were ~2–5 μm in length, approximately 200 nm in diameter, with an inner diameter of approximately 50 nm and a wall thickness of 50 nm. Capsicum-like PZS nanotubes were ~1–3 μm in length, with an outer diameter of 100–300 nm and an inner diameter of 20–50 nm. The main stem diameter of PZS branched nanotubes was approximately 200 nm, and the branched chain diameter was 20–100 nm. FTIR and XRD were used to characterize the chemical structure of PZSs, and the results are shown in Figure S2. There were no differences in the FTIR spectra or the XRD patterns of the PZSs with different morphologies.

Initially, we proposed that the formation mechanism of PZS with different morphologies occurred in the following manner: at the initial stage of the reaction at room temperature, the TEA catalyzed reaction between HCCP and BPS in THF was slow, forming oligomers and rod-like triethylamine hydrochloride (TEACl) crystals. PZS oligomers were spontaneously adsorbed onto the surface of the TEACl nanocrystals due to their low surface energy [13]. After washing with water, the TEACl dissolved and finally formed PZS nanotubes. The way that the reactants were added to the reaction solution influenced the resulting PZS morphology. For example, the slow and dropwise addition of the HCCP solution to the reaction solution resulted in the limited production of oligomers that were rapidly adsorbed onto one end of the TEACl nanocrystals. This allowed for easier adsorption of the subsequent oligomers onto the thicker end of the tube wall, and thereby, the capsicum-like PZS nanotubes were formed after the removal of TEACl. Similarly, when the HCCP solution was slowly added to the reaction in two steps, capsicum-like PZS nanotubes were formed in the primary growth period due to less oligomer production. A further addition of HCCP induced a new polymerization reaction, whereas new TEACl nanocrystals were formed on the surface of the original nanotubes. The branched PZS nanotubes were formed by secondary growth. PZS microspheres were formed during the high-temperature reaction where the primary particles collided with each other rapidly to form stable particles, and the stable particles gradually grew into spheres by absorbing oligomers from the solution.

### 3.2. Thermal Stability Analysis

**Figure 3:**
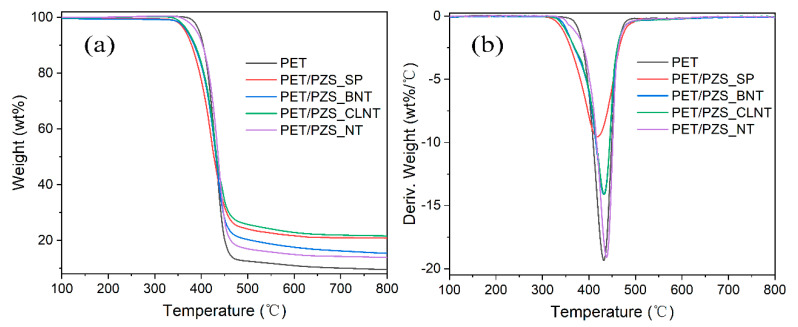
(**a**) TGA and (**b**) DTG curves of PET, PET/PZS_SP, PET/PZS_BNT, PET/PZS_CLNT, and PET/PZS_NT under a N_2_ atmosphere.

Previous studies have shown that the combustion behavior of polymers is, to a certain extent, correlated to the thermal degradation process. The thermogravimetry (TG) and derivative thermogravimetry (DTG) curves are shown in Figure 3 and the corresponding data are listed in Table 1, where T_5_ (the temperature at 5 wt% mass loss) is defined as the initial decomposition temperature and T_max_ represents the maximum degradation (or mass loss) rate. From Figure 3a, the PET/PZS composites have similar thermal degradation behaviors to PET. The initial degradation temperatures of the PET/PZS composites were all reduced relative to PET owing to the addition of the flame retardant material, with PET/PZS_SP exhibiting the lowest T_5_ of 366 °C. The flame retardant catalyzed and accelerated the degradation of PET, which is consistent with previously reported results in the literature [17,18]. The maximum degradation rate (T_max_) did not significantly change except for PET/PZS_SP, which was lower than that of PET. The higher the amount of residual carbon in the material at 800 °C, the more favorable it is for application in flame retardancy. According to the results of this study, PET had the lowest residual carbon at 800 °C (9.5 wt%). Notably, PZS_CLNT had the highest amount of residual carbon, followed by PZS_SP, which was unexpected. Among the PET/PZS composites, PZS_NT exhibited the lowest amount of residual carbon at 800 °C (13.8 wt%).

### 3.3. Analysis of Flame Retardancy and Burning Behavior

The dispersion of the flame retardant in the polymer influences the flame retardancy of the material, and the energy dispersive spectroscopy (EDS) mapping results in Figure S3 show that PZS with different morphologies are well dispersed in PET. LOI and UL-94 are commonly used as important indicators for evaluating flame retardancy of composites. The results of the LOI and UL-94 analysis for PET, PET/PZS_SP, PET/PZS_BNT, PET/PZS_CLNT, and PET/PZS_NT are displayed in Table 2. It was found that PET had the lowest LOI value (25.2 vol%), and the worst UL-94 grade (V-2 level), as well as a significant melt dripping phenomenon upon burning. The flame retardancy of the material improved after the addition of different morphologies of the PZS flame retardants. All the UL-94 levels reached the V-0 rating and the appearance of molten droplets was reduced. Among the composites investigated, PET/PZS_CLNT achieved the highest LOI value of 34.4 vol%, followed by PET/PZS_SP (33.1 vol%), then PET/PZS_BNT (32.8 vol%), and lastly, PET/PZS_NT (32.5 vol%). These results are consistent with the results obtained by thermal analysis of the residual carbon content of the composites at 800 °C.

**Figure 4:**
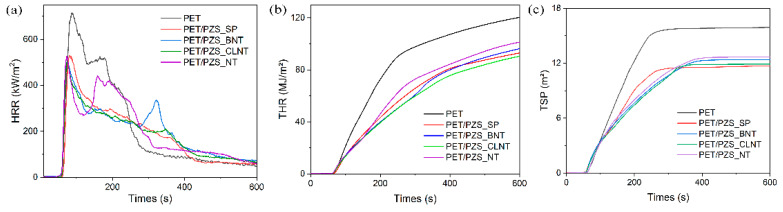
(**a**) HRR, (**b**) THR, and (**c**) TSP curves of pure PET, PET/PZS_SP, PET/PZS_BNT, PET/PZS_CLNT, and PET/PZS_NT.

The cone calorimeter that simulates the real combustion environment [19,20] was used to evaluate the burning behavior of pure PET and PET/PZS composites. The results of the heat release rate (HRR), total smoke production (TSP), and total heat release (THR) are shown in Figure 4 and the related data are summarized in Table 2. It was found that PET had a high peak heat release rate (PHRR) value of 715.94 kW/m^2^ and THR value of 120.3 MJ/m^2^. Both the PHRR and THR values decreased to a large extent relative to PET after the addition of flame retardants with different morphologies. Among the composites, PET/PZS_CLNT showed the best performance, with significant reductions in both the PHRR and THR values. Although the PHRR value of PET/PZS_SP was relatively high compared with the other flame retardants, its final THR value was relatively low. Among the composites, PET/PZS_NT exhibited the poorest flame retardant performance. The flame retardancy results were consistent with the LOI results. Notably, the PET TSP value was 15.9 m^2^, indicating that it produces a large amount of smoke upon burning. After the addition of flame retardants with different morphologies, the TSP values were significantly lower compared with that of PET. It is noteworthy that PET/PZS_CLNT and PET/PZS_SP released the least amount of smoke upon burning. These results indicate that PET/PZS_CLNT and PET/PZS_SP not only exhibit improved flame retardancy, but also have a significant smoke suppression effect.

### 3.4. Pyrolysis Products Analysis

**Figure 5:**
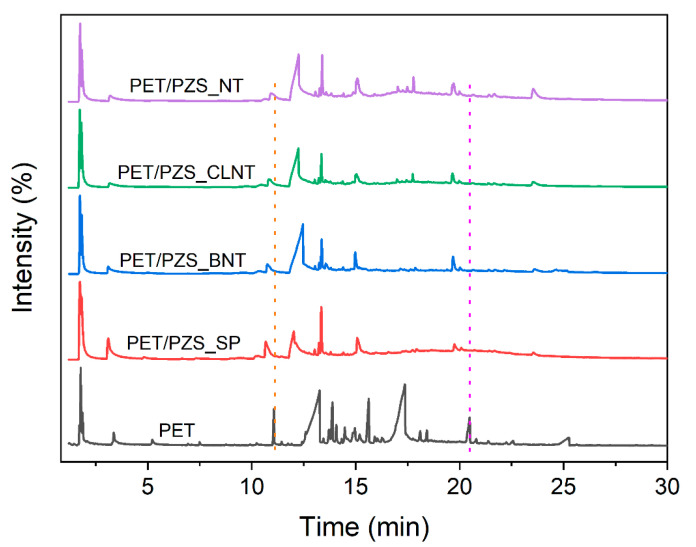
Py-GC/MS of PET and PET/PZS composites at 600 °C.

The analysis of the cracking products of PET and its composites aids in understanding and elucidating the flame retardant mechanism. The Py-GC/MS curves of PET, PET/PZS_SP, PET/PZS_BNT, PET/PZS_CLNT, and PET/PZS_NT are shown in Figure 5, and the MS data are summarized in Table 3. Many PET cracking peaks were observed, which indicates that many kinds of substances are cracked when PET is combusted. The number of cracking peaks decreased after the addition of flame retardants with different morphologies compared with PET. Moreover, the cracking peaks of the same substances were advanced, indicating that the flame retardant could catalyze the pyrolysis of PET. These results are consistent with the TGA results. Furthermore, the non-combustible gas, CO_2_, produced by cracking, drastically increased from 5.27% for PET to 16.38% and 18.06% for PET/PZS_CLNT and PET/PZS_SP, respectively. The addition of flame retardants led to a reduction in the content of combustible aromatic compounds, such as benzene. Furthermore, the release of a large amount of non-combustible gas and a reduction in the content of combustible substances inhibited the combustion of the substrate.

### 3.5. Condensed and Gas Phase Analysis

**Figure 6:**
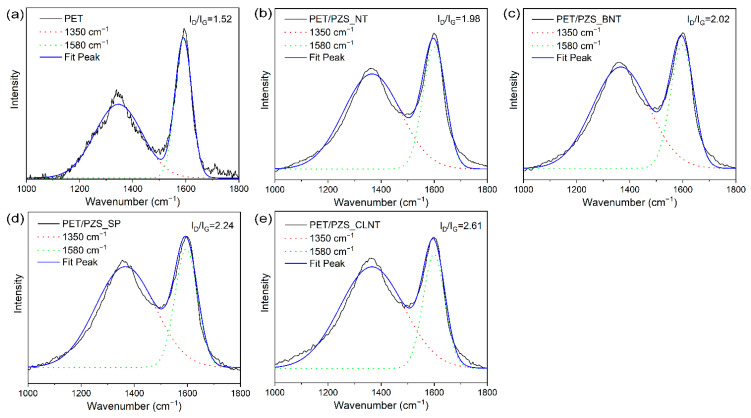
Raman spectra of the char residues for (**a**) PET, (**b**) PET/PZS_NT, (**c**) PET/PZS_BNT, (**d**) PET/PZS_SP, and (**e**) PET/PZS_CLNT.

To investigate the condensed phase flame retardant mechanism, the char residues were analyzed using Raman and X-ray photoelectron spectroscopy (XPS). The Raman spectra of the char residues for PET, PET/PZS_SP, PET/PZS_BNT, PET/PZS_CLNT, and PET/PZS_NT are shown in Figure 6. The I_D_/I_G_ value, which is defined as the area ratio of the D band (1350 cm^−1^) to the G band (1580 cm^−1^) [21,22], is often used to evaluate the microcrystal size of the char layer [23,24,25]. Higher I_D_/I_G_ values represent better flame retardant properties [24,26]. PET had the lowest I_D_/I_G_ value, and the I_D_/I_G_ values of the flame retardants with different morphologies, which were higher than that of PET, had the following order: PET/PZS_CLNT > PET/PZS_SP > PET/PZS_BNT > PET/PZS_NT, which indicates that PET/PZS_CLNT can form a highly compact and cohesive carbon layer during combustion. The compact and cohesive carbon layer can form a barrier that inhibits mass and heat transfer during combustion, thus inhibiting combustion [27,28].

**Figure 7:**
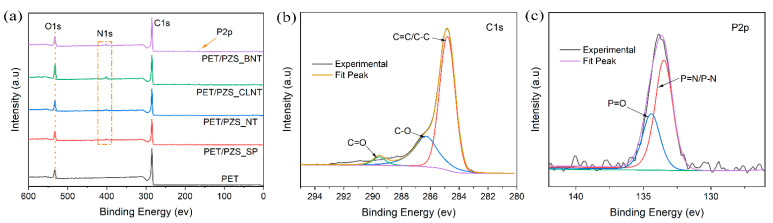
(**a**) XPS survey scan profiles, (**b**) C1s, and (**c**) P2p core spectra of the char residues for PET/PZS_CLNT.

To further investigate the relationship between residual carbon and flame retardancy, the residual carbon elements of PET and its composites were studied using XPS. Figure 7 shows the XPS profiles of the residual carbon of PET, PET/PZS_SP, PET/PZS_BNT, PET/PZS_CLNT, and PET/PZS_NT. It was found that the PET residual carbon contains C and O elements, whereas the flame retardant composites contained N and P elements in addition to C and O. From the C1s spectra of Figure 7b, the binding energy peak at 289.5 eV, 286.3 eV, and 284.3 eV were attributed to C=O, C-O, and C=C/C-C, respectively [29,30,31]. Based on the results, it was clear that the main carbon in the residual carbon was in the form of C=C and/or C-C. In Figure 7c, the P2p core spectrum of PET/PZS_CLNT was divided into two peaks. The first peak at 134.5 eV was attributed to P=O bonds due to the phosphorous compounds produced in air during the combustion process, and the other peak was attributed to P=N/P-N groups [32,33,34]. These results provide strong evidence that PZS functioned in the condensed phase during the combustion process by forming a stable phosphate ester carbon layer [35,36].

**Figure 8:**
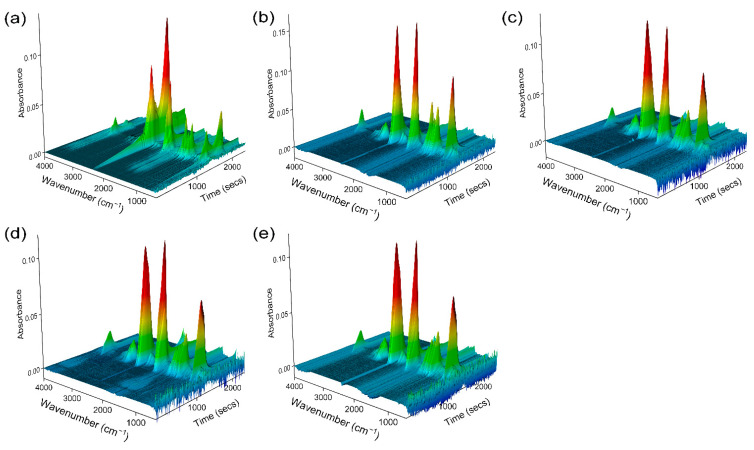
3D TG-IR spectra of (**a**) PET, (**b**) PET/PZS_NT, (**c**) PET/PZS_BNT, (**d**) PET/PZS_SP, and (**e**) PET/PZS_CLNT.

**Figure 9:**
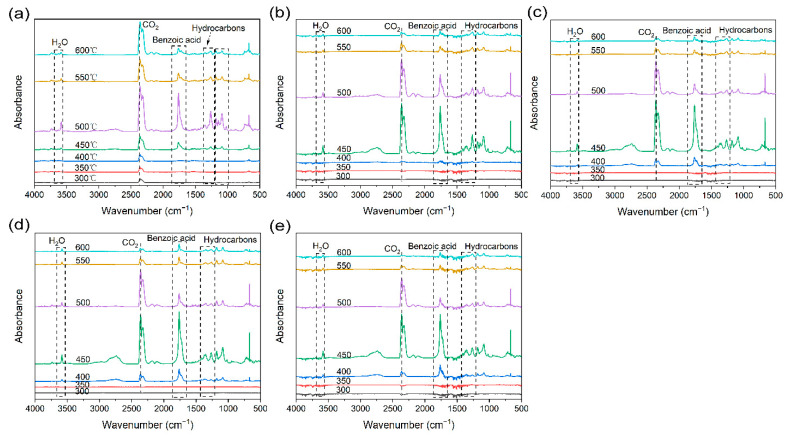
FTIR spectra of pyrolysis products at different temperatures for (**a**) PET, (**b**) PET/PZS_NT, (**c**) PET/PZS_BNT, (**d**) PET/PZS_SP, and (**e**) PET/PZS_CLNT.

To further understand the flame retardant mechanism, the products generated by the cracking process were analyzed using TG-IR. The 3D TG-IR and FTIR spectra of the products released from the thermal cracking process of PET, PET/PZS_SP, PET/PZS_BNT, PET/PZS_CLNT, and PET/PZS_NT are shown in Figure 8 and Figure 9, respectively. Overall, the PET/PZS composites released similar gaseous products as PET. Several gaseous products were detected within the range of 1040–1175 cm^−1^ (aliphatic ethers), 1230–1450 cm^−1^ (hydrocarbons), 1760 cm^−1^ (benzoic acid), 2360 cm^−1^ (CO_2_), and 3550–3600 cm^−1^ (H_2_O) [37,38,39]. It is evident that the amount of CO_2_ and benzoic acid released after the addition of the flame retardant was highest at 450 °C, whereas for PET it was highest at 500 °C. This indicates that the flame retardant catalyzes the cleavage of PET, which is consistent with the TGA results. Only a small amount of CO_2_ and benzoic acid was released at 600 °C, whereas a large amount was released from PET. This may be due to the formation of a highly dense and continuous char layer, owing to the addition of a flame retardant, which inhibits the release of cracking products [40].

## 4. Conclusions

In this study, four different morphologies of PZS were successfully synthesized in one solvent (THF). PZS nanotubes were prepared by a room temperature reaction of HCCP with BPS in THF. By increasing the reaction temperature, PZS gradually transformed from nanotubes into microspheres. The dropwise addition of the reactants yielded capsicum-like and branched PZS nanotubes. Different morphologies of PZS flame retardants were added into PET to form separate PET/PZS composites, which all had improved flame retardancy. From highest to lowest, the flame retardancy of the composites followed the order: PET/PZS_CLNT > PET/PZS_SP > PET/PZS_BNT > PET/PZS_NT. To investigate the effect of morphology on flame retardancy, Py-GC/MS, Raman, XPS, and TG-IR were used to elucidate the mechanism of flame retardants. The compact and coherent carbon layer and the release of non-combustible gas played an important role in the flame retardancy of the PET/PZS composites; therefore, this study provided a facile synthetic method for morphology-controlled nanomaterials and presented a novel strategy for the design of new flame retardant materials.

## Figures and Tables

**Table 1:** TGA data of PET, PET/PZS_SP, PET/PZS_BNT, PET/PZS_CLNT, and PET/PZS_NT.

Samples	T_5_ (°C)	T_max_ (°C)	Char Residue at 800 °C (wt%)
PET	395	433	9.5
PET/PZS_SP	366	416	20.8
PET/PZS_NT	390	438	13.8
PET/PZS_CLNT	372	432	21.5
PET/PZS_BNT	370	432	15.4

**Table 2:** LOI, UL-94, and cone calorimeter results for PET, PET/PZS_SP, PET/PZS_BNT, PET/PZS_CLNT, and PET/PZS_NT.

Samples	LOI (vol%)	UL-94		PHRR (kW/m^2^)	THR (MJ/m^2^)	TSP (m^2^)
		Rating	Dripping			
PET	25.2	V-2	Severe	715.94	120.3	15.9
PET/PZS_SP	33.1	V-0	Slow	530.74	93.0	11.7
PET/PZS_NT	32.5	V-0	Slow	525.68	101.3	12.7
PET/PZS_CLNT	34.4	V-0	Slow	506.28	90.5	11.9
PET/PZS_BNT	32.8	V-0	Slow	504.22	96.3	12.4

**Table 3:** Pyrolysis products of PET and PET/PZS composites under 600 °C.

Peak No.	Main Products	Pure PET		PET/PZS_SP		PET/PZS_BNT		PET/PZS_CLNT		PET/PZS_NT	
		Time (min)	Intensity (%)	Time (min)	Intensity (%)	Time (min)	Intensity (%)	Time (min)	Intensity (%)	Time (min)	Intensity (%)
1	CO_2_	1.772	5.27	1.733	18.06	1.734	12.09	1.724	16.38	1.743	13.89
2	CH_3_CHO	1.866	1.61	1.807	18.77	1.817	12.16	1.807	16.51	1.831	8.05
3	C_6_H_6_	3.365	1.78	3.1	12.71	3.098	6.3	3.174	5.09	3.193	6.45
6	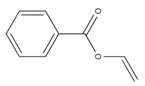	11.068	2.6	10.676	8.38	10.746	4.5	10.825	5.32	10.946	5.31
9	C_6_H_5_COOH	13.263	28.36	12.021	21.45	12.463	43.16	12.248	36.11	12.253	37.92
11	C_6_H_5_CHCHCOOCHCH_2_	13.711	1.65	13.227	1.41	13.245	0.82	13.222	0.82	13.257	0.88
12	C_6_H_5_-C_6_H_5_	13.881	3.98	13.345	7.74	13.364	5.26	13.349	4.76	13.385	4.95
13	C_6_H_5_(COOCHCH_2_)_2_	15.628	5.17	15.079	5.96	14.977	3.39	15.036	4.24	15.081	6.56
19	C_15_H_12_O_3_	18.427	0.93	17.939	0.81	17.87	0.64	17.745	1.37	17.786	2.03
31	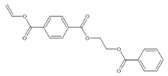	25.255	3.51	23.558	1.16	23.602	1.69	23.539	3.07	23.544	5.95

## Data Availability

The data presented in this study are available on request from the corresponding author.

## Supplementary Materials

The following supporting information can be downloaded at: https://www.mdpi.com/article/10.3390/polym14102072/s1, Figure S1: SEM images of PZS obtained at different temperatures: (a) room temperature, (b) 80 °C, and (c) 120 °C; Figure S2: (a) FTIR and (b) XRD profiles of HCCP, BPS, PET/PZS_SP, PET/PZS_BNT, PET/PZS_CLNT, and PET/PZS_NT; Figure S3: EDS mapping of (a) PET/PZS_SP, (b) PET/PZS_CLNT, (c) PET/PZS_BNT, and (d) PET/PZS_NT.
