# Supplementary material for: Morphology-Controlled Synthesis of Polyphosphazene-Based Micro- and Nano-Materials and Their Application as Flame Retardants
Source: Polymers (Basel). 2022 May 19;14(10):2072. doi: 10.3390/polym14102072 (PMC9144817; doi:10.3390/polym14102072)
Supplement: Supplementary file 1 [file polymers-14-02072-s001.zip › polymers-1688772-supplementary.pdf]

Article

Morphology-Controlled Synthesis of Polyphosphazene-Based Micro- and Nano-Materials and Their Application as Flame Retardants

Yuanzhao Zhu [1,2], Wei Wu [1,2], Tong Xu [1,2], Hong Xu [1,2], Yi Zhong [1,2], Linping Zhang [1,2], Yimeng Ma [1,2], Xiaofeng Sui [1,2], Bijia Wang [1,2], Xueling Feng [1,2] and Zhiping Mao [1,2,3,4,*]

1 Key Laboratory of Science and Technology of Eco-Textile, Ministry of Education, College of Chemistry, Chemical Engineering and Biotechnology, Donghua University, Shanghai 201620, China; 1189171@mail.dhu.edu.cn (Y.Z.); wuwei@dhu.edu.cn (W.W.); xutong.868@163.com (T.X.); hxu@dhu.edu.cn (H.X.); zhongyi@dhu.edu.cn (Y.Z.); zhang_lp@dhu.edu.cn (L.Z.); yimeng.ma@dhu.edu.cn (Y.M.); suixf@dhu.edu.cn (X.S.); bwang@dhu.edu.cn (B.W.); xlfeng@dhu.edu.cn (X.F.)

2 Innovation Center for Textile Science and Technology of Donghua University, Donghua University, Shanghai 201620, China

3 National Engineering Research Center for Dyeing and Finishing of Textiles, Donghua University, Shanghai 201620, China

4 National Manufacturing Innovation Center of Advanced Dyeing and Finishing Technology, Donghua University, Shanghai 201620, China

* Correspondence: zhpmao@dhu.edu.cn

S1. Characterization of PZSs

Figure S1 shows the resulting product morphologies obtained at different temperatures: (a) room temperature, (b) 80 ℃, and (c) 120 ℃.

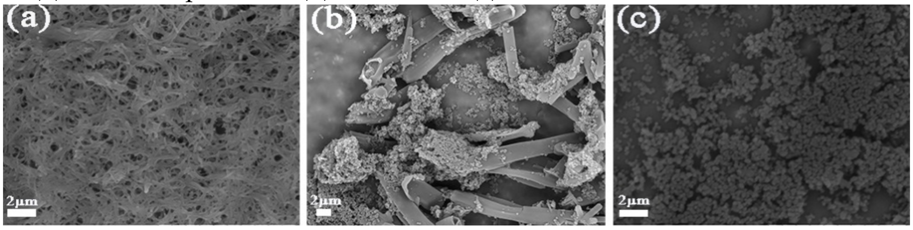

Figure S1. SEM images of PZS obtained at different temperatures: (a) room temperature, (b) 80 ℃, and (c) 120 ℃.

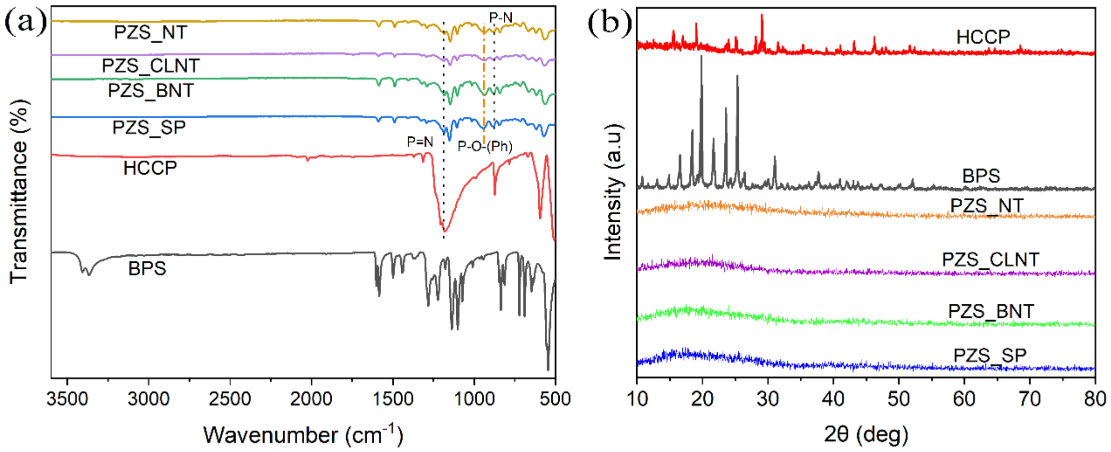

Figure S2. (**a**) FTIR and (**b**) XRD profiles of HCCP, BPS, PET/PZS_SP, PET/PZS_BNT, PET/PZS_CLNT, and PET/PZS_NT.

S2. Dispersibility of PZS in PET

According to the energy dispersive spectroscopy (EDS) mapping of PET/PZS_SP, PET/PZS_CLNT, PET/PZS_BNT, and PET/PZS_NT, PZS with different morphologies is well dispersed in PET.

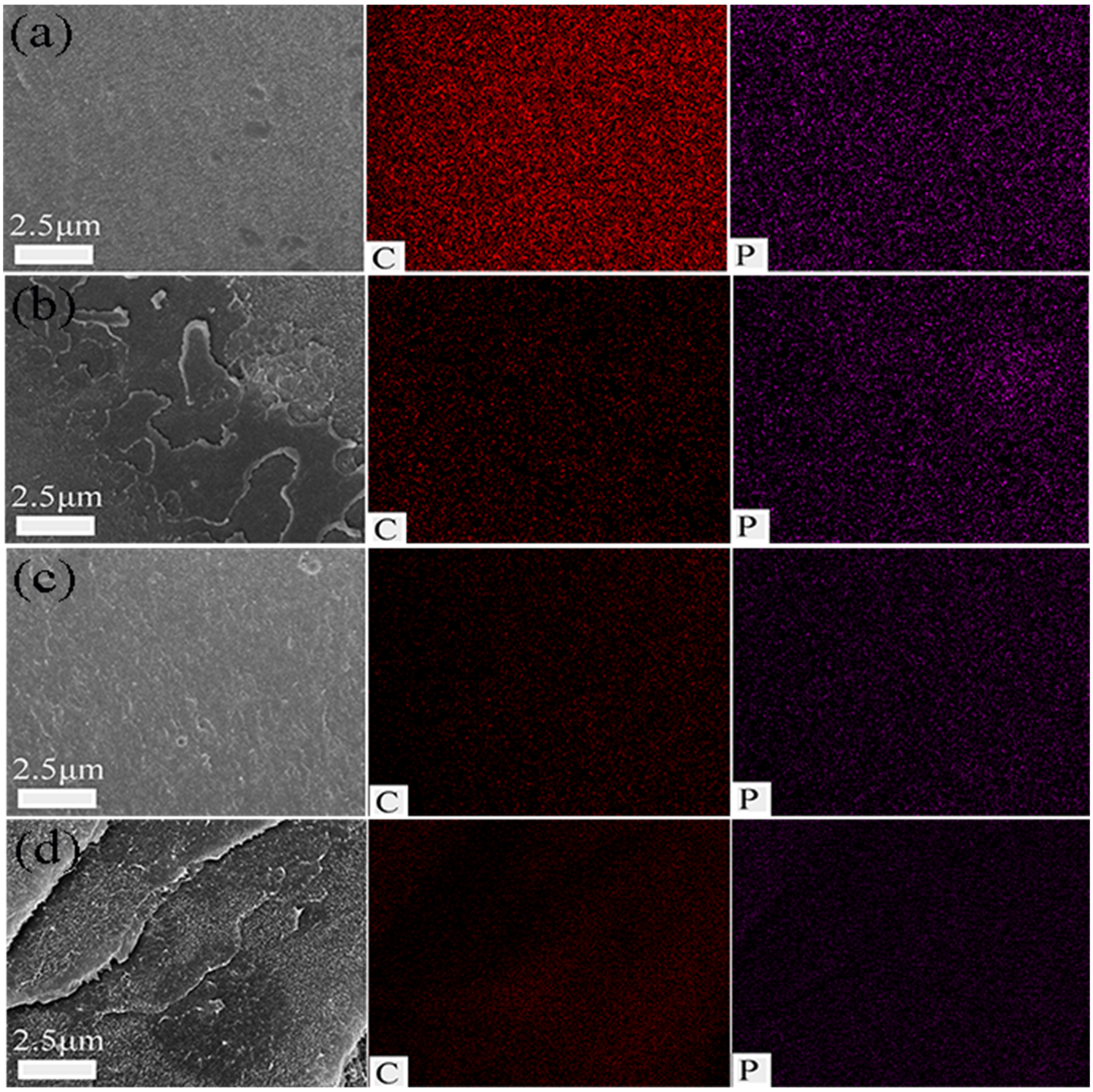

Figure S3. EDS mapping of (**a**) PET/PZS_SP, (**b**) PET/PZS_CLNT, (**c**) PET/PZS_BNT, and (**d**) PET/PZS_NT.
